# Supplementary material for: Rubisco forms a lattice inside alpha-carboxysomes
Source: Nat Commun. 2022 Aug 18;13:4863. doi: 10.1038/s41467-022-32584-7 (PMC9388693; doi:10.1038/s41467-022-32584-7)
Supplement: Supplementary file 1 — Supplementary Information [file 41467_2022_32584_MOESM1_ESM.docx]

**Supplementary Figures and Tables**


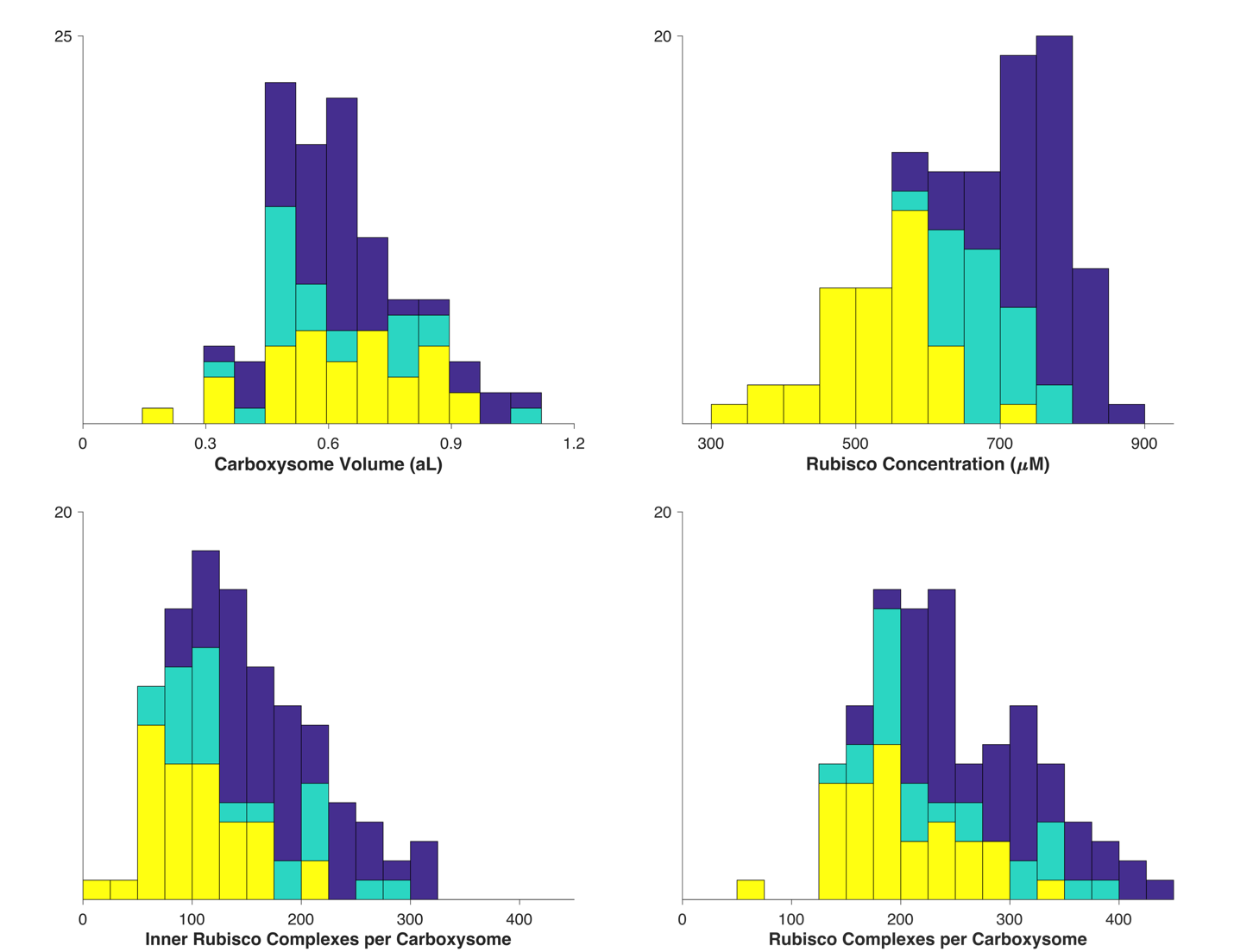


**Supplementary Figure 1**: Carboxysome parameters. Top left: volume per CB. Top right: Rubisco concentration per CB. Bottom: Rubisco complexes per CB. Inner Rubisco (left) excludes shell-adjacent Rubisco. Yellow, sparse; teal, dense; indigo, ordered.

| **Sample** | **Ordered** | **Dense** | **Sparse** |
| --- | --- | --- | --- |
| Purified CBs | 49/107 | 23/107 | 35/107 |
| *H. neapolitanus* cells | 38/158 | 119/158 | 1/158 |

**Supplementary Table 1: Proportion of ordered, dense and sparse CBs in each dataset.**


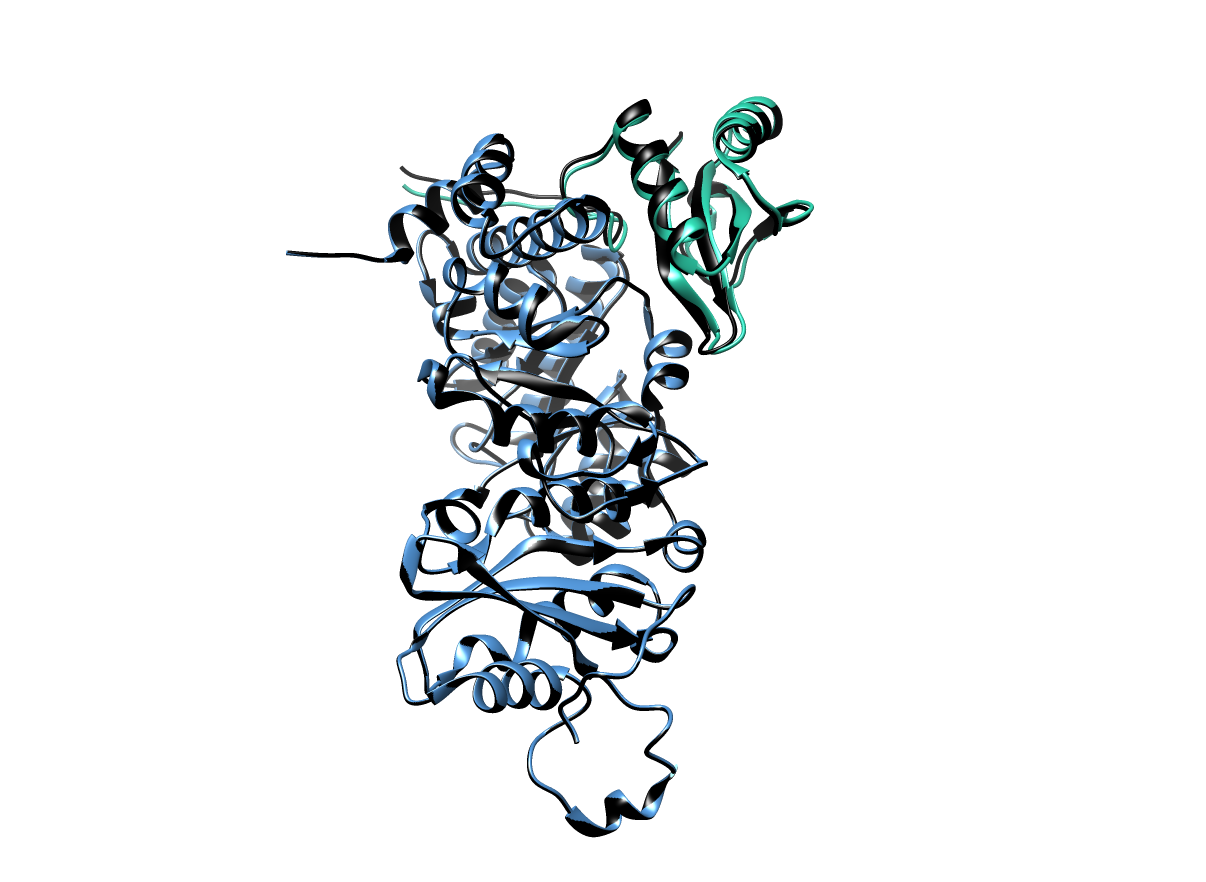


**Supplementary Figure 2**: Orientation of the Rubisco subunits inside the CB*.* Black: 1SVD crystal structure. Color: large (blue) and small (green) subunits docked independently in our map.


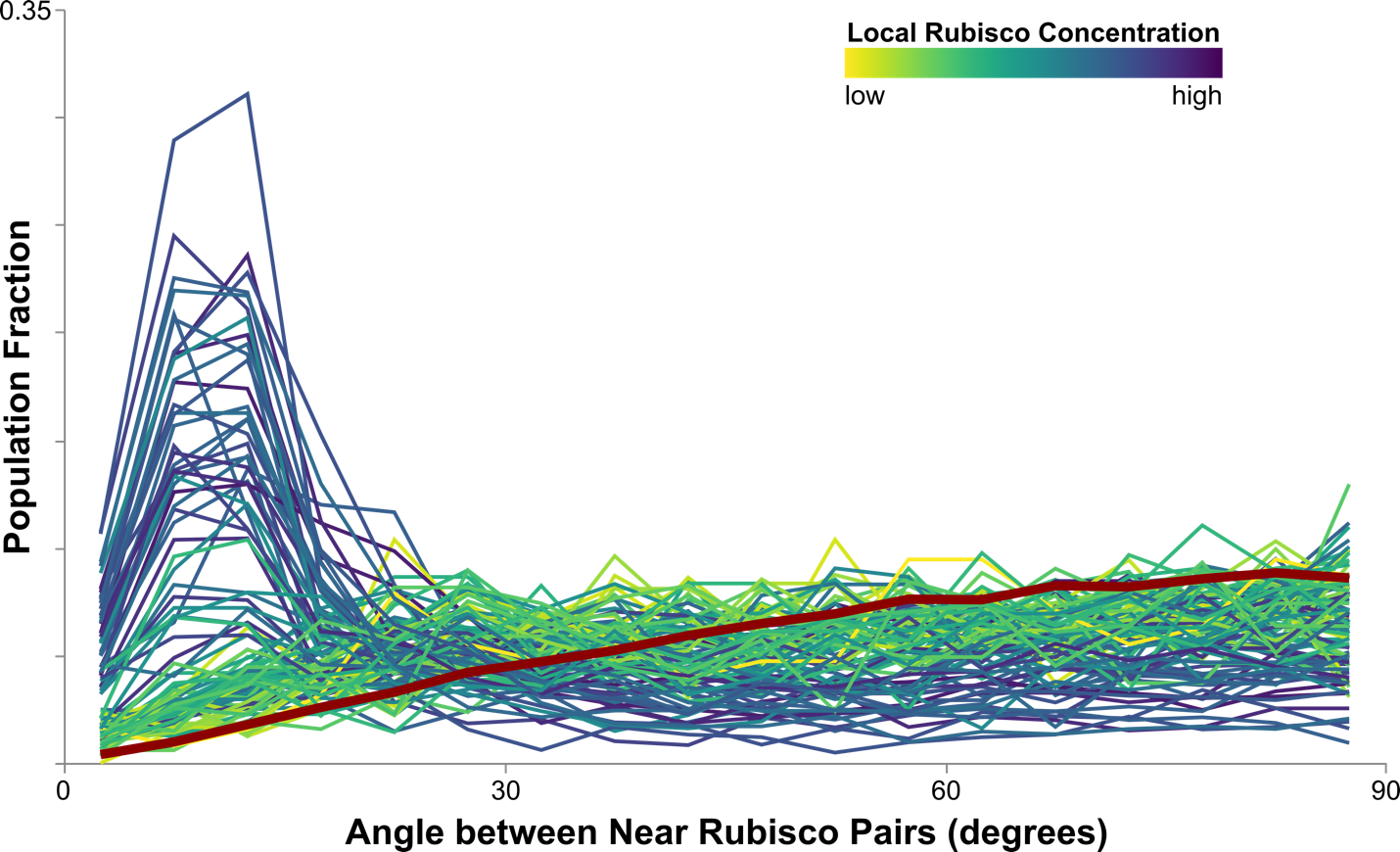


**Supplementary Figure 3**: Histogram tracings of nearest-neighbor angle alignment inside carboxysomes. Line points are the centers of 5-degree bins. At low Rubisco concentrations, nearest neighbors have random orientation (red line). As concentration increases, Rubisco begins to align at a low nearest-neighbor angle.


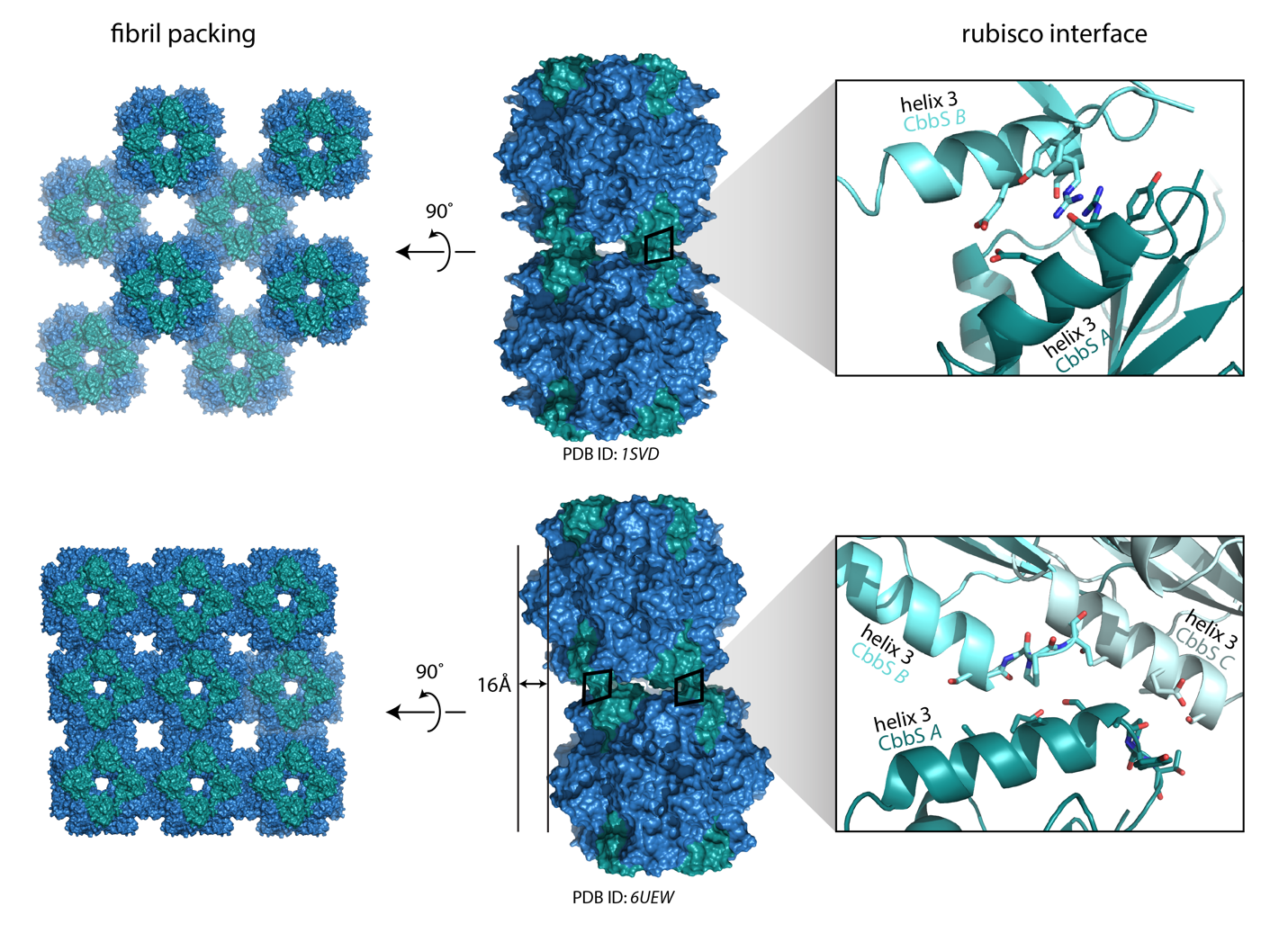


**Supplementary Figure 4**: Longitudinal Rubisco fibril interfaces in crystal structures of Form 1A Rubisco (*H. neapolitanus*) from the Protein Data Bank. Blue, large subunit; green, small subunit. Both interactions are mediated by small subunit helix 3, but neither shows a specific, strong interaction between side chains.


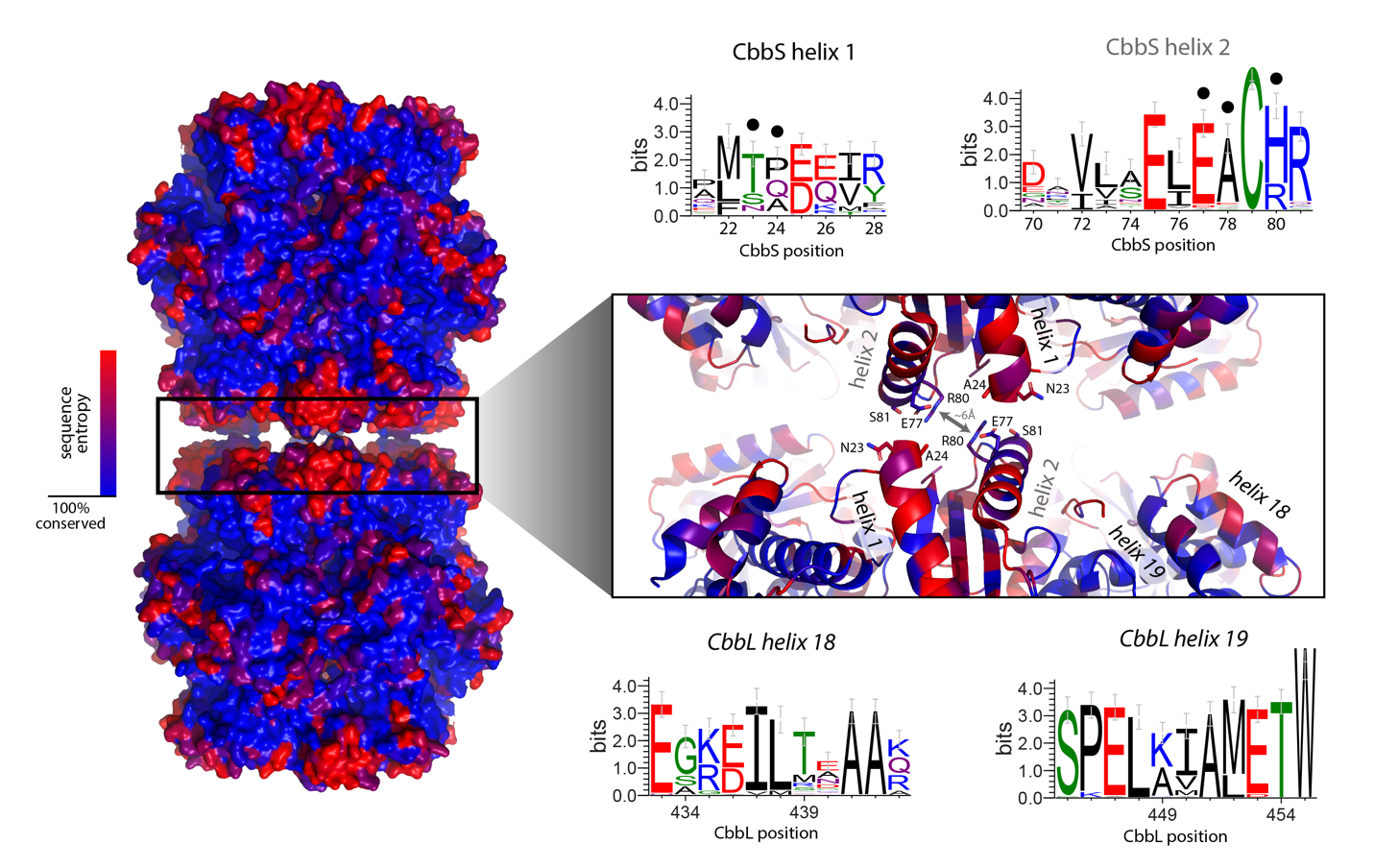


**Supplementary Figure 5:** Surface representation of the modeled Rubisco-Rubisco interface. Coloring is by calculated amino acid sequence entropy (blue, 100% conservation). Inset: Zoomed in view of interface and potential contacts at CbbS helices 1 and 3. Also shown for comparison are CbbL helices 18 and 19 that do not participate in Rubisco-Rubisco binding.


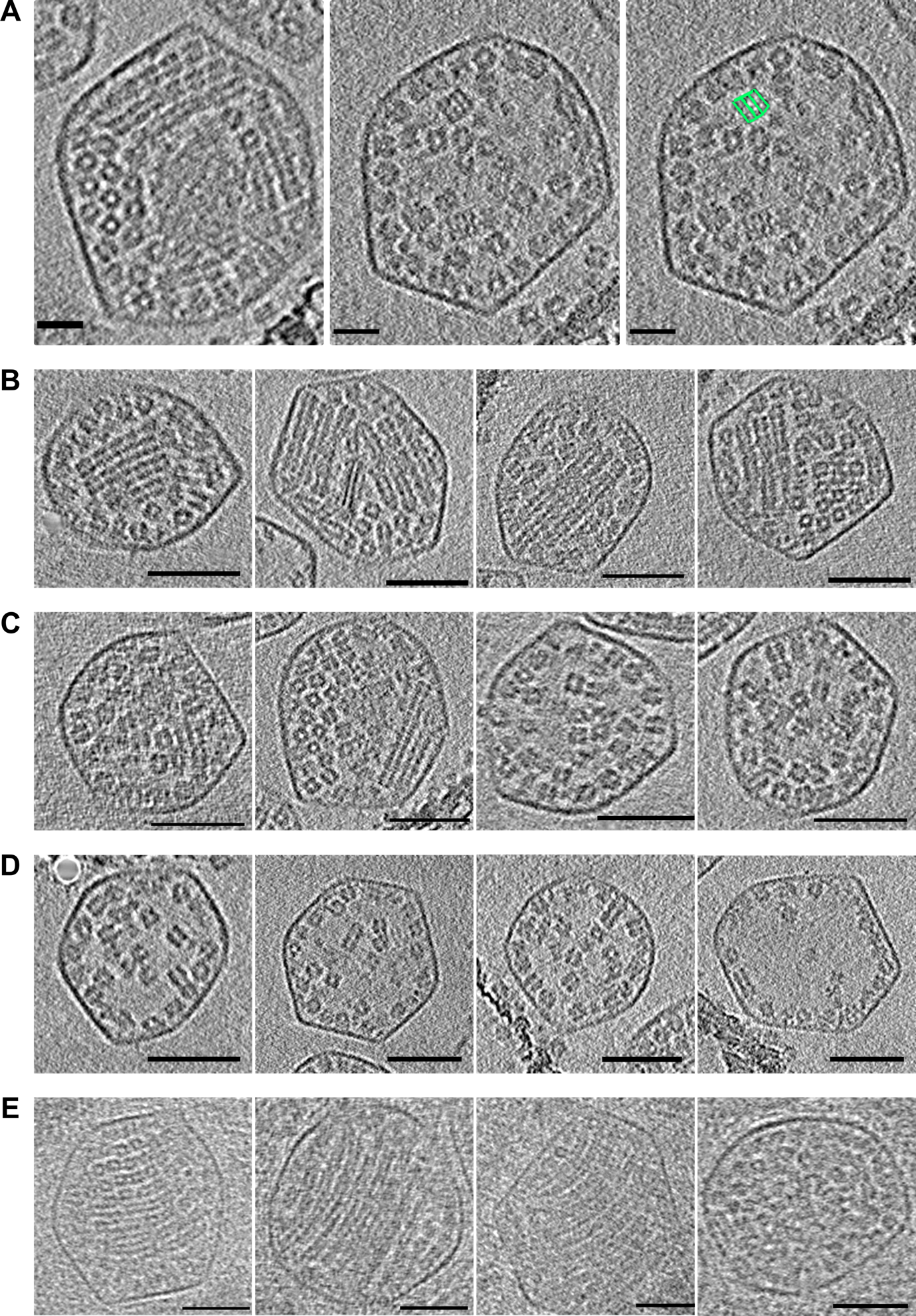


**Supplementary Figure 6**: heterogeneity in carboxysomes. A: Left: an 8.6 nm orthoslice through a CB with fibrils arranged along the shell. The center has a dense, poorly resolved area that be a disordered peptide aggregate. Center: a 4.3 nm orthoslice through a CB containing two visible large non-Rubisco complexes. Right: one complex segmented in green. Scale bar 20 nm. B: additional orthoslices of ordered CBs; two have single central lattices and two have multiple lattices along the CB sides. C: additional orthoslices of dense CBs; two contain fibrils but lack a full lattice. D: additional orthoslices of sparse CBs. E: additional orthoslices of CBs within *H. neapolitanus* cells. Three are ordered; one is dense. Scale bars in B-E are 50 nm.


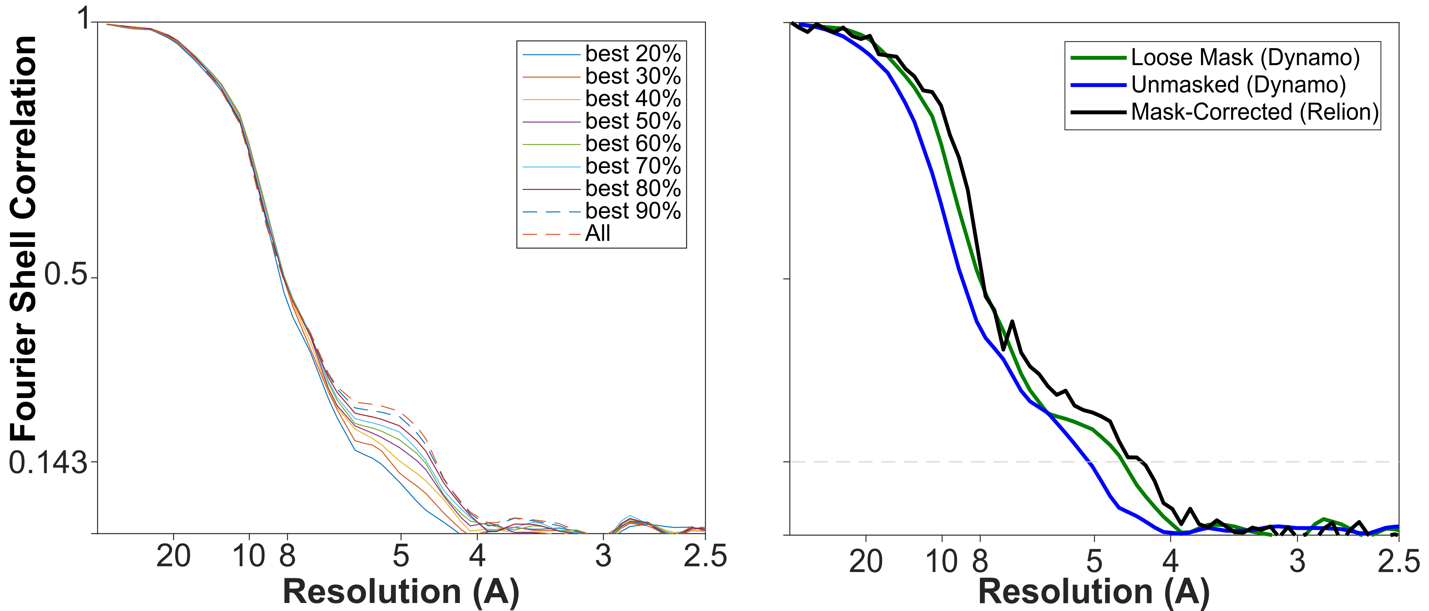


**Supplementary Figure 7**: Left: Effect of particles on resolution. Removing the worst-correlating fraction of particles decreases resolution. Right: Fourier Shell Correlation for the final Rubisco subtomogram average. Unmasked FSC, blue; loose masked FSC, green; mask-corrected FSC, black. The resolution at 0.143 (grey dashed line) is 4.5 Å.

|  | **Carboxysomes** | **Cells** |
| --- | --- | --- |
| Number of tomograms | 62 | 41 |
| Microscope | Titan Krios G3 | Titan Krios G3 |
| Nominal defocus range | -1 to -5.5 μm | -3.5 to -8 μm |
| Nominal pixel size | 1.104 Å | 2.153 Å |
| Tilt range | -66:66 degrees | -60:60 degrees |
| Tilt increment | 3 degrees | 3 degrees |
| Tilt method | Dose-symmetric | Dose-symmetric |
| Electron dose per tilt | 2.9 e^-^/Å^2^ | 3 e^-^/Å^2^ |
| Total electron dose | 130 e^-^/Å^2^ | 120 e^-^/Å^2^ |

**Supplementary Table 2. Structural data collection parameters.**
