# Supplementary material for: Rubisco forms a lattice inside alpha-carboxysomes
Source: Nat Commun. 2022 Aug 18;13:4863. doi: 10.1038/s41467-022-32584-7 (PMC9388693; doi:10.1038/s41467-022-32584-7)
Supplement: Supplementary file 3 — Description of Additional Supplementary Files [file 41467_2022_32584_MOESM3_ESM.pdf]

Description of Additional Supplementary Files

File Name: Supplementary Movie 1
Description: The Rubisco lattice of an ordered carboxysome. The movie tilts and rotates the carboxysome lattice displayed in Figure 3B. Rubiscos are rendered as rectangles with length to scale and width decreased for visual clarity. Fibrils containing 3 or more Rubisco complexes are opaque; all other Rubiscos are transparent.
