# Supplementary material for: Rubisco forms a lattice inside alpha-carboxysomes
Source: Nat Commun. 2022 Aug 18;13:4863. doi: 10.1038/s41467-022-32584-7 (PMC9388693; doi:10.1038/s41467-022-32584-7)
Supplement: Supplementary file 5 — Reporting Summary [file 41467_2022_32584_MOESM5_ESM.pdf]

nature portfolio

Corresponding author(s): Jensen, GJ
Metskas, LA

Last updated by author(s): Jul 28, 2022

Reporting Summary

Nature Portfolio wishes to improve the reproducibility of the work that we publish. This form provides structure for consistency and transparency in reporting. For further information on Nature Portfolio policies, see our Editorial Policies and the Editorial Policy Checklist.

Statistics

For all statistical analyses, confirm that the following items are present in the figure legend, table legend, main text, or Methods section.

n/a	Confirmed	
☐	☒	The exact sample size (*n*) for each experimental group/condition, given as a discrete number and unit of measurement
☐	☒	A statement on whether measurements were taken from distinct samples or whether the same sample was measured repeatedly
☒	☐	The statistical test(s) used AND whether they are one- or two-sided<br>*Only common tests should be described solely by name; describe more complex techniques in the Methods section.*
☒	☐	A description of all covariates tested
☐	☒	A description of any assumptions or corrections, such as tests of normality and adjustment for multiple comparisons
☐	☒	A full description of the statistical parameters including central tendency (e.g. means) or other basic estimates (e.g. regression coefficient) AND variation (e.g. standard deviation) or associated estimates of uncertainty (e.g. confidence intervals)
☒	☐	For null hypothesis testing, the test statistic (e.g. F, t, r) with confidence intervals, effect sizes, degrees of freedom and P value noted<br>*Give P values as exact values whenever suitable.*
☒	☐	For Bayesian analysis, information on the choice of priors and Markov chain Monte Carlo settings
☒	☐	For hierarchical and complex designs, identification of the appropriate level for tests and full reporting of outcomes
☒	☐	Estimates of effect sizes (e.g. Cohen's *d*, Pearson's *r*), indicating how they were calculated

Our web collection on statistics for biologists contains articles on many of the points above.

Software and code

Policy information about availability of computer code

Data collection	SerialEM (v. 3.8.0 beta, 3.9.0 beta), Digital Micrograph (v. 3.31.2162.0, 3.43.3213.0)
Data analysis	IMOD (v. 4.9.8), ctffind4 (v. 4.1.13, 4.1.10, 4.1.8), Matlab R2017b, novaCTF (no version number), dynamo (v. 1.1.333, 1.1.514), Relion (SBGrid environment, v. 3.0, 3.1), UCSF Chimera (v. 1.12), custom code (https://observablehq.com/collection/@lametskas/cbpaper, https://github.com/trogon44/carboxysome_tomography/tree/master/rubisco_conservation, https://github.com/trogon44/carboxysome_tomography/tree/master/rubisco_density)

For manuscripts utilizing custom algorithms or software that are central to the research but not yet described in published literature, software must be made available to editors and reviewers. We strongly encourage code deposition in a community repository (e.g. GitHub). See the Nature Portfolio guidelines for submitting code & software for further information.

Data

Policy information about availability of data

All manuscripts must include a data availability statement. This statement should provide the following information, where applicable:

- Accession codes, unique identifiers, or web links for publicly available datasets
- A description of any restrictions on data availability
- For clinical datasets or third party data, please ensure that the statement adheres to our policy

Rubisco subtomogram average unfiltered half-maps, full filtered map, mask and FSC curves have been deposited in the EMDB (accession number EMD-27654). The full set of subtomograms used in this study is available in EMPIAR, along with sample tomograms and their frames (accession number 11125). Raw tomogram movie frames may be accessed through the Caltech Electron Tomography Database (https://etdb.caltech.edu/, search for author Metskas).

We used publicly available Rubisco structures from H. neapolitanus in this study, available through the Protein Data Bank: 1SVD (https://www.rcsb.org/structure/1SVD) and 6UEW (https://www.rcsb.org/structure/6uew).

Field-specific reporting

Please select the one below that is the best fit for your research. If you are not sure, read the appropriate sections before making your selection.

☒ Life sciences ☐ Behavioural & social sciences ☐ Ecological, evolutionary & environmental sciences

For a reference copy of the document with all sections, see nature.com/documents/nr-reporting-summary-flat.pdf

Life sciences study design

All studies must disclose on these points even when the disclosure is negative.

Sample size	Dataset size was determined by collecting as many tomograms as possible in a 3-day microscope session; all collected data was used. This study has 32,930 identified Rubisco complexes in 139 full, intact carboxysomes found in 62 tomograms. Ultrastructure and organization analysis used a subset containing 26,224 Rubisco complexes in 107 carboxysomes. For the in vivo dataset, sample size was determined by the number of tomograms collected over a 2-day microscope session. We collected 41 usable tomograms of intact H. neapolitanus cells, which contained 158 carboxysomes where Rubisco orientations were visible.
Data exclusions	No data were excluded from the subtomogram averaging. Ultrastructure analysis excludes tomograms collected at too low defocus for high-accuracy particle identification (< 15% false positive/negative rates). Cellular tomography was used only for visual comparison with the purified dataset (Ext. Table 1, Figure 6, Ext. Figure 6E).
Replication	For the purified carboxysome dataset, one dataset is reported here and was the third technical replicate (other replicates discarded due to vibrations caused by defective clipping supplies). A screening dataset was collected with a second biologically distinct sample and was consistent with the data reported here. For the cellular tomograms, one dataset is reported here and is the sole technical replicate. Screening datasets were collected on two biologically distinct samples and were consistent with the data reported here.
Randomization	Biological randomization was not necessary for a structural study. Carboxysomes were allocated into even/odd subsets for structural analysis according to their assigned ID number, which was given based on spatial order (left to right, bottom to top, tomogram to tomogram).
Blinding	Blinding was not possible because the investigator assigns the carboxysome ID number, but IDs are assigned prior to structural analysis so the investigator would not be able to discriminate. In general, blinding is not considered necessary for a structural investigation where particles are assumed to be identical.

Reporting for specific materials, systems and methods

We require information from authors about some types of materials, experimental systems and methods used in many studies. Here, indicate whether each material, system or method listed is relevant to your study. If you are not sure if a list item applies to your research, read the appropriate section before selecting a response.

Materials & experimental systems

n/a	Involved in the study	
☒	☐	Antibodies
☒	☐	Eukaryotic cell lines
☒	☐	Palaeontology and archaeology
☒	☐	Animals and other organisms
☒	☐	Human research participants
☒	☐	Clinical data
☒	☐	Dual use research of concern

Methods

n/a	Involved in the study	
☒	☐	ChIP-seq
☒	☐	Flow cytometry
☒	☐	MRI-based neuroimaging
